# Astrocyte Heterogeneity in Regulation of Synaptic Activity

**DOI:** 10.3390/cells11193135

**Published:** 2022-10-05

**Authors:** Anna Kruyer

**Affiliations:** Department of Neuroscience, Medical University of South Carolina, Charleston, SC 29425, USA; kruyer@musc.edu

**Keywords:** astrocytes, astroglia, synapse, heterogeneity, plasticity

## Abstract

Our awareness of the number of synapse regulatory functions performed by astroglia is rapidly expanding, raising interesting questions regarding astrocyte heterogeneity and specialization across brain regions. Whether all astrocytes are poised to signal in a multitude of ways, or are instead tuned to surrounding synapses and how astroglial signaling is altered in psychiatric and cognitive disorders are fundamental questions for the field. In recent years, molecular and morphological characterization of astroglial types has broadened our ability to design studies to better analyze and manipulate specific functions of astroglia. Recent data emerging from these studies will be discussed in depth in this review. I also highlight remaining questions emerging from new techniques recently applied toward understanding the roles of astrocytes in synapse regulation in the adult brain.

## 1. Introduction

Early studies on astroglia noted both their morphological complexity and heterogeneity [1]. Modern research continues to expand upon these original observations, noting profound heterogeneity in astrocyte structure and function both within and between brain regions. As many as 16 astrocyte or related glial subtypes have been identified according to morphofunctional analyses, with an additional three subtypes identified only in humans [2,3,4]. Likely complexity exists within each of these subgroups, increasing the possibility of highly specialized astroglial types in the healthy adult brain.

Accordingly, recent studies using transcriptomic profiling have identified five astrocyte subtypes in the mouse cortex and hippocampus alone, each with unique spatial positioning [5]. When the authors of this study compared the top ten genes expressed by each of these astroglial types, only a single gene was shared between subtypes. Moreover, transcription factors were prominent among genes that were differentially expressed by astrocyte types, underscoring the value of this strategy in identifying markers for these newly identified cell groups. These analyses also indicate that while certain capacities may be shared across astrocyte subtypes, at least in the cortex, including glycolysis, lactate production, cholesterol metabolism, and glutamate catabolism; transcriptomic profiles consistent with gliotransmission, immune responsiveness, and blood–brain barrier regulation are unique to some astroglial types but not others, suggesting that there may be specific types that perform unique functions within the brain [5].

Advances in monitoring astrocytes have given rise to additional approaches useful for identifying astroglial subtypes. For example, between brain regions, astrocytes have been shown to exhibit different patterns of calcium flux as well as different degrees of synaptic adjacency [6]. These observations are not only suggestive of different degrees and/or mechanisms of synapse regulation by astrocytes between brain regions, but have led to genesis of new tools and strategies for targeting and manipulating unique astroglial populations [7,8,9]. Together, these approaches provide us with the ability to examine in more detail how pathogenesis in various disease states alters function of specific astroglial populations.

## 2. Origins of Astrocyte Heterogeneity

The existence of spatially patterned, transcriptionally unique astroglial types in the adult brain (as in [5,6,7,8,9,10]) suggests that astrocyte heterogeneity may derive, at least in part, from unique cell lineages that determine their molecular and functional profiles in adulthood [11]. Alternatively, many have argued that astrocyte heterogeneity, and even transcriptional profiles of astroglial types, derives from the neurovascular landscape where they find themselves positioned in the brain [4,12,13,14,15]. As mentioned above, a number of molecular markers, including those critical for energy metabolism, are broadly shared by astrocytes, even across subtypes [5]. Instead, transcriptional profiles associated with unique astrocyte subtypes may be driven by recruitment of unique transcription factor combinations through triggers in the local extracellular milieu. In an attempt to address this fundamental question, Clavreul et al. performed lineage tracing on astroglia in the mouse cortex over the course of gliogenesis, and found that, despite sharing a common progenitor, astrocytes in the cortex diverge from the layered pattern observed by neurons and exhibit structural variability despite sharing a common lineage [12]. The authors conclude that astrocyte size and complexity are determined at a single cell level and that, while astrocytes within the cortex derive from glial progenitors pre and postnatally, these cells migrate and proliferate, and ultimately mature based on cues in the local environment, and not strictly as a consequence of lineage [12]. Indeed, culturing astrocytes in the presence of neurons increases their complexity and heterogeneity, inducing expression of hundreds of genes and leading to production of proteins essential for synapse regulation, including GAT-3 and excitatory amino acid transporters (EAATs) GLAST and GLT-1 [16]. Studies in adult rodents also point to astrocyte heterogeneity deriving from local neural signals. For example, astrocytes in the somatosensory cortex of the mouse brain respond with calcium flux to stimulation of layer 4 neurons projecting to layer 2/3, suggesting that astrocytes can be tuned to different neural circuits [17]. In subcortical structures like the dorsolateral ventral pallidum, astrocytes respond by changing their synaptic association in response to cues predicting heroin delivery according to their anteroposterior positioning in the structure, suggesting heterogeneity within the structure, perhaps deriving from the spatial segregation of active inputs [18]. Thus, a reasonable conclusion is that astrocyte heterogeneity, both between cells and within cellular microdomains, derives from signals released by nearby neurons, the local vasculature, and other glia.

## 3. Morphological Heterogeneity of Astroglia and Relevance for Synapse Regulation

Important roles for astroglia in synaptic regulation have long been appreciated. Astrocytes express glutamate and GABA transporters and signal using gliotransmitters to regulate synaptic transmitter release [19]. Astrocytes also exhibit morphological plasticity, adjusting their synaptic adjacency in response to neural activity, presumably to regulate synaptic and extrasynaptic actions of released neurotransmitters. Astrocytes also provide metabolic support to neurons [20], buffer extracellular potassium essential for maintaining the neuronal membrane potential [21], supply substrates for production of glutamate and GABA in neurons [22], participate in redox homeostasis [23], and recruit blood flow in response to neural activity [24]. Although not discussed in much depth here, astrocytes also promote synapse formation and pruning (see [4] for detailed descriptions of such mechanisms). Given the many important roles for astrocytes at synapses, their adjacency to neurons, and particularly to synapses, has received much attention as a dynamic and functionally critical way that astrocytes alter neural activity [19].

Between brain regions, it appears that astrocytes differ dramatically in their patterns of synaptic insulation. For example, astrocytes exhibit a bias toward synapses in the hippocampus, where each astrocyte surrounds an average of four neuronal somata and roughly 100,000 synapses [6,25]. Instead, striatal astrocytes cover a larger territory on average, but due to reduced density of excitatory synapses, encompass roughly half as many synapses as in the hippocampus [6,25,26]. Within a single brain region as well, astrocytes exhibit different patterns of adjacency with different synapse types. For example, astrocytes in the dorsal striatum are more closely apposed to glutamatergic compared with dopaminergic terminals, maintaining an average of ~50 nm distance from the former and ~250 nm distance from the latter [27]. In all, astrocytes appear to remain tightly apposed (<10 nm) to roughly half of striatal synapses, but more than 100 nm away from nearly 20% of the remaining synapses [27]. What functions astrocytes can perform at these distances is a matter of debate. Certainly, gliotransmission and transmitter uptake are likely to be more effective when astrocytes are immediately adjacent to synapses, however astrocytes can also perform a number of extrasynaptic functions, including signaling at extrasynaptic receptors on neurons. For example, the cystine–glutamate exchanger, expressed on astroglia, maintains extracellular glutamate levels in the nucleus accumbens core, part of the ventral striatum, and this is thought to provide tone on presynaptic inhibitory metabotropic glutamate receptors (mGluRs) that attenuate transmitter release [28]. Astrocyte protrusions ~100–200 nm from the synaptic cleft may also serve to suppress transmitter spillover from reaching receptors on nearby portions of the neuron, such as neighboring spines. It has also been noted that astrocytes are more closely associated with post-synaptic compared with pre-synaptic neuronal elements [29], but whether this metric is heterogeneous and/or plastic has yet to be shown.

In the nucleus accumbens core, like in the dorsal striatum, astrocytes exhibit a range of synaptic adjacency when measured at the level of each astrocyte [30] and like in the dorsal striatum, astrocytes exhibit a bias in their adjacency with different synaptic subtypes [9]. For example, in adult rats, the degree of confocal coregistration between the astroglial membrane and D_2_ receptor-expressing postsynaptic neurons is nearly double that compared with D_1_-positive synapses [9], with likely consequences on synapse-selective regulation by astroglia. The factors that determine the “set point” of astrocyte synapse adjacency is an exciting field of study, and likely involves differences in receptor type and density expression in astroglial microdomains. That mGluR signaling on astroglia has been linked with motility of astroglial filapodia supports this hypothesis [31]. For example, denser expression of mGluRs linked with phosphorylation of ezrin, an actin-binding protein selectively expressed in perisynaptic astroglial processes, would serve to maintain very near-adjacent astroglial coverage, given sufficient synaptic activity. Instead, synaptic inactivity would have the opposite effect, resulting in reduced levels of ezrin phosphorylation and synaptic association by astroglial processes when extracellular glutamate levels are relatively low. Notably, activity at excitatory synapses has been linked with both increases [30,32,33,34] and decreases [35,36,37] in astrocyte-synapse adjacency in in vitro, ex vivo and in vivo studies, with astrocytes appearing to both extend toward, and retract from active synapses, respectively. Notably, astrocyte retraction from synapses during excitatory synaptic activity has been shown to permit glutamate spillover and recruitment of neighboring synapses, amplifying the effects of excitatory transmission [36]. The reasons for these apparently contradictory findings are unknown, but might again involve unique receptor expression within whole astroglia or at astroglial microdomains. For example, while mGluR3 or 5 stimulation on astroglia has been linked with ezrin phosphorylation and fine process extension [31], stimulation of the Na^+^-K^+^-2Cl^−^ cotransportor NKCC1 is required for astrocyte perisynaptic process retraction in the hippocampus in response to neural activity [36]. Astrocyte fine process retraction has also been linked to function of the gap junction protein connexin 30, since loss of connexin 30 expression in the hippocampus leads to increased synaptic insulation of GLT-1-positive astroglial processes that suppress synaptic activity [38]. Thus, differential expression of various receptors and transporters may shape astrocyte morphological responses to synaptic activity. This is an intriguing idea, since synaptic insulation by astroglia has dramatic effects on synaptic potentiation and depression (see Section 3.1 below for details), raising the possibility that changes in expression of a single receptor or transporter could “flip” an astrocyte’s perisynaptic response to transmitter release, with potentially dramatic consequences on synaptic potentiation and/or recruitment.

Local signals driving morphological features of astroglia are an important corner of the literature that remains relatively under-explored, and whether astroglial subtypes derive their structure in response to different signal cascades in different brain regions is not yet known. Regardless, Khakh et al. have proposed that a more precise lexicon be used for discussions of astroglial morphology, with “processes” growing increasingly imprecise for description of astroglial protrusions that approach synapses (leaflets), blood vessels (endfeet), as well as the primary and secondary branches from which these protrusions extend [13]. Identification of proteins selectively expressed or engaged in motility of these different astroglial components would strongly support such a nomenclature and a clearer delineation of the function of these cellular subregions.

Despite vast heterogeneity in synaptic regulation by astroglia, the degree to which astrocytes support synaptic potentiation vs. depression appears to depend on the brain region, with astrocytes participating in synaptic depression more often in the striatum compared with the hippocampus, for example (see Table 1). The various signaling modalities that astrocytes use to potentiate or depress synapses are described in the subsequent section. Despite this, spines are often reported to be larger when perisynaptic astroglia are present, although larger spines also have a greater perimeter absent of astroglial membrane from which synaptically released transmitter can escape the synaptic cleft [39]. In the mature brain, Witcher et al. propose that new synapses form more easily where astroglia are absent, perhaps as a consequence of transmitter spillover [39]. Moreover, astroglial presence at large spines is explained as a consequence of increased transmitter release that both recruits astroglial protrusions and increases spine head diameter [39]. Synaptogenesis stimulated by development or neurodevelopment-like triggers (i.e., cocaine [40]) may require astroglial signaling [41], perhaps contributing to other reports that indicate closer adjacency of astroglia at thin spines [33,42]. For example, astrocytes exhibit more near-adjacent association with thin spines compared with mushroom spines in the hippocampus in 8-week old rodents [42], but are more likely to be present at mushroom spines, albeit at a relatively further distance [6], perhaps suggesting different types of signaling between the two types of spines and astroglia.

As mentioned above, striatal astrocytes also exhibit morphological differences in their adjacency to different striatal subcircuits, with a higher degree of synaptic adjacency at D_2_ receptor-expressing neurons in the ventral striatum [9], but not dorsal striatum [27]. If astrocytes decrease synaptic activity at D_2_-synapses according to mechanisms presented in Table 1, then their increased insulation of this synapse type suggests tighter regulation of activity within this circuit at baseline. Interestingly, operant training can alter these patterns of synapse adjacency, where extinction of a reward-cue association triggers retraction of astrocytes from D_2_-synapses [9]. Accordingly, this retraction would be predicted to reduce astrocyte-dependent inhibitory control of D_2_-synapse activity, permitting suppression of reward-seeking, a response specifically mediated by D_2_-expressing neurons [88,89]. These discoveries add a layer of complexity to the discussion of astrocyte heterogeneity, as native heterogeneity appears to be further complicated by salient life experiences. Thus, on top of impressive heterogeneity at baseline, environmental and pharmacological triggers can elicit plasticity in unique astroglial subtypes in order to regulate neural circuit function. It is also well known that astrocyte motility in various brain structures depends on zeitgeber time [90,91], adding another dimension to the impressive heterogeneity encountered in studies on astrocyte function.

### 3.1. Heterogeneity in Gliotransmission and Neurotransmitter Transport

That astrocytes exhibit heterogeneity in their responses to activity within different neural subcircuits was first demonstrated in the dorsal striatum, where astrocytes were shown to exhibit calcium flux and undergo circuit-selective signaling with neurons of either the direct or indirect pathways [78]. The dorsal striatum is composed mainly of spiny neurons that project either directly to the substantia nigra or indirectly to the nigra via the external segment of the globus pallidus and the subthalamic nucleus [92], and astrocytes release glutamate in response to neural activity that stimulates NMDA receptors on homotypic neuron pairs [78]. Despite the apparent distinction between astroglial subtypes that segregate their adjacency and activity according to neuronal populations, individual astrocytes encompass somata from multiple classes of neurons [27]. Instead, astrocytes are likely tuned to respond to different patterns of neural activity via distinct mechanisms. Covelo et al. showed that distinct stimulation parameters at hippocampal Schaffer collaterals trigger either synaptic potentiation, via astroglial glutamate release, or heterosynaptic depression via astroglial ATP release [93]. Thus, a single astrocyte is capable of different responses following distinct patterns of neural activity at a single synapse.

Perhaps given this apparent segregation of astroglia according to neural subcircuits and the far-reaching implications of changes in astrocyte-synapse adjacency as discussed above, members of the Khakh lab have developed a tool for high throughput analysis of astroglial adjacency to different neural subtypes [27]. When applied in dorsal striatum, astrocytes exhibit preference for different synapse types, with FRET signal, indicative of <10 nm adjacency, highest at thalamic inputs, followed by collateral, cortical and nigral terminals. Although the function(s) of astrocytes in regulating activity of these synapse types has yet to be fully characterized, this structural organization suggests enhanced regulatory control over thalamic signaling, relative to cortical and nigral [27]. This, paired with the increased insulation of excitatory vs. dopaminergic synapses by astrocytes in dorsal striatum, suggests that at baseline, astrocytes play a greater role in regulating glutamatergic compared with. dopaminergic signaling, perhaps due to their chief role in glutamate uptake. While the same pattern has not been parsed out in ventral striatum, accumbens astrocytes do appear to play a critical role in regulating glutamatergic signaling in response to dopamine inputs. Astrocytes respond to dopamine release via their own D_1_ receptor expression and release ATP/adenosine that dampens glutamate release through its action on presynaptic A_1_ receptors [77]. Whether astrocytes detect dopamine from a distance, or are heterogeneous in their insulation of dopaminergic terminals, despite a low overall proximity, is not known.

Application of DREADD technology to study various G-protein signaling cascades in astrocytes has illuminated further flexibility on the part of astrocytes regarding their capacity to respond to certain input types. G_q_-coupled DREADDs have been linked to a host of astroglial responses in various brain regions and behavioral paradigms. G_q_-coupled signaling in the striatum alone has been linked to astrocyte fine process extension, astrocyte release of glutamate via exocytosis, as well as ATP/adenosine signaling [9,54,94]. Outside of the striatum, G_q_-DREADD stimulation in vivo is also linked with various behavioral consequences of both increased and decreased synaptic activity. To illustrate the diverse consequences of G_q_ signaling in astrocytes, Table 2 shows a variety of outcomes linked to astrocyte G_q_ stimulation in various brain regions and disease models. Whether the responses in these examples derive from the use of designer, rather than endogenous receptors, or whether astrocytes respond to GPCR stimulation via diverse responses in individual brain nuclei is not well understood. Note also that classic signaling cascades linked to various GPCRs in neurons may not be strictly upheld in astroglia. For example, the G_i/o_-coupled CB1 receptor has been shown to couple with G_z_, G_q_, and G_12/13_ in astroglia [95,96], perhaps giving rise to the diversity of astroglial responses following activation of G_q_ signaling (Table 2).

There is notable heterogeneity over the course development and by brain region in expression of neurotransmitter transporters, such as GLT-1, GLAST and GAT-3 [136]. Glutamate uptake via transporters like GLT-1 is considered to be a fundamental function of astrocytes [137]. However, GLT-1 expression and function are perhaps also heterogenous within and between brain regions, and certainly throughout neurodevelopment [137,138]. In the nucleus accumbens, astrocytes can be stratified not only according to their total levels of GLT-1 expression, but also by the cellular localization of GLT-1 [139]. Although GLT-1 is downregulated in nucleus accumbens astrocytes after use of and withdrawal from addictive drugs [140], heroin (but not sucrose) use and withdrawal not only increases astrocyte adjacency to D_1_ receptor-expressing synapses in the ventral striatum and at the terminals of these cells in the ventral pallidum [9,18], but also increases the proportion of astroglia with GLT-1 targeted extrasynaptically [9]. Both of these adaptations, the insulation of D_1_-synapses, and the increase in extrasynaptic GLT-1, are thought to negatively regulate drug seeking behavior, by depressing activity at D_1_-synapses [9,78], and by preventing synaptic recruitment via glutamate spillover between synapses [36]. Extrasynaptic levels of GLT-1 are further increased by cues that reinstate drug seeking [9]. GLT-1 surface diffusion is known to be incredibly dynamic [139] and may be regulated by metabotropic glutamate receptors, much like fine process motility [31,101]. Interestingly, astrocytes that increase their surface expression of GLT-1 during cued relapse remain retracted from synapses, and astrocytes that undergo morphological plasticity during cued relapse express relatively low levels of GLT-1, perhaps indicating that different patterns of mGluR expression may contribute to genesis of these functional “types” [9].

It is evident that Ca^2+^ signals regulate a diversity of astroglial functions in the brain, including regulation of blood flow [141], morphological plasticity [34,142], and various types of gliotransmission [77,78,143] Ca^2+^ signaling in astrocytes also appears to be a rather heterogeneous, with microdomains in a single astrocyte responding uniquely [144,145,146,147]. Ca^2+^ flux in astrocyte microdomains can happen apparently spontaneously, without requiring neuronal firing, and can also occur in response to neurotransmitter release in branches and branchlets, as well as in astrocyte somata potentially occurring in response to different stimuli and with diverse consequences [124,146,148,149,150]. Broad calcium signals that appear uniform across multiple cells have also been observed during volumetric release of neuromodulators [144,151,152]. However, whether different astroglia exhibit different patterns of calcium activity, or have different capacities to signal using calcium has not been clarified. For a more thorough discussion of function and heterogeneity of astroglial Ca^2+^ signals, see [8,13,153].

### 3.2. Astrocyte Heterogeneity in Disease States

The extent to which reactive astrogliosis, the astrocytic response to disease and/or injury, disrupts astrocytic roles in synaptic regulation likely depends on many factors, as reactive astrocytes are themselves heterogeneous [154]. In spinal cord cultures, addition of a cytokine cocktail alters signaling kinetics at GABAergic synapses, but the degree to which these alterations involve acquired or disrupted astroglial functions has not been shown [155]. Initiation of pro-inflammatory cytokine signaling in astroglia can trigger transcriptional shifts, resulting in downregulation of proteins thought to be involved in detecting and responding to synaptic activity, such as GPCRs [156]. It has been proposed that these transcriptional shifts may be linked with behavioral and cognitive alterations observed in CNS disease, such as sleep disturbance and social withdrawal [156]. Thus, while in some cases, environmental insults can trigger programmed increases in astrocyte complexity, it more often appears to be the case that psychiatric and neurocognitive illnesses are characterized by a collapse of astrocyte heterogeneity required for synapse regulation in key brain regions. For example, in a number of different neurodegenerative disorders, astrocytes undergo downregulation of the potassium channel Kir4.1, contributing to neuronal dysfunction [157]. Broad transcriptomic analysis of astroglial gene expression in mouse models of Huntington’s disease and in human patients also demonstrates a general downregulation of important astroglial functional markers [7]. In major depressive disorder, a disease where substantial data from human post-mortem brain tissue illustrates involvement of astroglia in both disease onset and severity as well as in treatment-assisted recovery [158,159,160], astroglial atrophy has been observed without classic indications of reactivity [161,162]. Reductions in astroglial size and/or number have been noted in cortical and limbic regions in patients with mood disorders [163]. Likewise downregulation of classic astroglial functional markers have been noted in animal models of major depressive disorder, including aquaporins, connexins, glutamate transporters and glutamine synthetase [163]. Following traumatic brain injury, reductions in GAT-3 expression coincident with onset of reactive astrogliosis, disrupt cognitive function, supporting the notion that astrocyte activation interrupts functional astrocyte heterogeneity by conversion to a reactive state [118].

Likewise in animal models of substance use disorders, downregulation of glutamate transporters in the ventral striatum coincides with loss of glutamate homeostasis [140], reduction in astroglial volume [30,164] and a general reduction in the degree of variability in synaptic association across populations of astrocytes [9,30]. This loss in astrocyte morphological complexity has been noted in other models characterized of addiction vulnerability [165,166], but is not noted after operant training with a non-pathological reinforcer such as sucrose [30]. Since synaptic insulation and glutamate transporter expression in the ventral striatum have both been shown to be protective features of astrocytes that attenuate drug seeking in rodents, loss of complexity in both of these measures appears to be a pathological adaptation induced by drug experience [167,168,169].

## 4. Harnessing Heterogeneity

Awareness of the degree to which astrocytes exhibit heterogeneity is likely to help scientists develop more careful studies and to interpret results with appropriate caution. It is notable that in a majority of studies on astroglia where viruses are used to drive astroglial expression of various markers or alter gene expression, astrocytes being studied express GFAP, the main promoter used to label and manipulate astrocytes. It is likely that application of tools that make use of different promoters will uncover further astrocyte heterogeneity, since GFAP itself is expressed in a heterogeneous manner and is observed to be up or downregulated in various disease models [154,170]. Lack of GFAP promoter expression in different brain regions results from chromatin condensation, and thus GFAP expression may define a distinct subset of derivates from a common astrocyte precursor lineage [170]. Notably, astrocyte labeling according to GFAP expression delineates subregions not previously identified, lending weight to the value of structural analyses in assessment of astroglial subtypes [171].

The emergence of new data on astrocyte heterogeneity also raises exciting questions regarding whether and how to harness this knowledge to develop better tools for preclinical studies. For example, are there unique populations of astrocytes capable of responding via astrogliosis or does reactive astrogliosis simply prevent astrocytes from engaging in the variety of essential functions needed for normal brain health and function? That is, does astrogliosis represent a type of astroglial heterogeneity, or does it simply limit native heterogeneity required for synaptic homeostasis? Clarifying this point is an important goal that will aid in designing studies to determine how specific pathologies may disrupt certain astroglial functions, and illuminating ways to restore normal physiology in disease states. Finally, do astrocytes couple within and between types, and can researchers use astroglial coupling to target astrocyte subpopulations? Or instead, should we focus our energy on clarifying molecular markers for different astroglial types to understand their relevance to health and disease? Emerging data are beginning to answer these fundamental questions. For example, in amygdala, a morphologically unique astrocyte subpopulation expressing the oxytocin receptor are not interconnected, but largely couple via gap junctions to astroglia that do not express the oxytocin receptor [172].

Lastly, to what extent are our preclinical discoveries on astrocyte heterogeneity relevant to humans, or do new and unexplored rules apply concerning human astroglia? It is worth noting that on average, human astrocytes cover roughly twice the diameter of brain tissue as compared with rodent astrocytes, and contact up to 2 million synapses, compared with rodent astrocytes that are reported to contact up to 120,000 synapses [2,173]. These comparisons suggest that the capacity for morphological complexity is far greater in human compared with rodent astrocytes. There also appear to be astroglial types present in humans that are not found in rodents, including interlaminar and varicose astrocytes characterized by long projection-like processes that reach deep cortical layers [2,173,174]. Likewise, it remains a possibility that certain astroglial functions in humans will not be observed in rodents. Identifying ways to interrogate astrocyte functional diversity in the human brain during development and aging, and in disease, remains a top priority in the field.

## Figures and Tables

**Table 1:** Reported astrocyte functions at synapses in regions of the adult brain. While astrocytes do undergo signaling to potentiate and recruit synapses (orange), many mechanisms of astroglial signaling described in the literature serve to attenuate synaptic activity (blue), particularly outside of the hippocampus. mAChR, metabotropic acetylcholine receptor; SICs, slow inward currents; NMDAr, NMDA receptor; mGluR, metabotropic glutamate receptor; AMPAr, AMPA receptor; CBr, cannabinoid receptor; LTP, long-term potentiation; LTD, long-term depression.

Brain Region	Astroglial Protein	Gliotransmitter	Neuronal Effect	Ref.
**Cortex**	--	ATP/adenosine	reduced synaptic activity via presynaptic A_1_ receptors	[43]
	CB1	glutamate	spike-timing dependent depression via presynaptic NMDArs	[44]
	--	ATP/adenosine	attenuated synaptic activity through downregulation of GABAB receptors	[45]
	--	ATP/adenosine	NMDAr downregulation via P2X receptors to increase threshold of LTP induction	[46]
	Kir4.1, Cx43	--	potassium spatial buffering to attenuate network excitability	[47]
	GLT-1	--	reduced synaptic potentiation	[48]
	--	--	spatial blockade of glutamate spillover and synaptic recruitment	[36]
	IGF-1	ATP/adenosine	LTD at cortical synapses	[49]
	mAChR	glutamate	SICs	[50]
	--	ATP/adenosine	neural synchronization	[51]
	mAChR	D-serine	local field potentials characteristic of slow wave sleep	[52]
**Hippocampus**	--	ATP/adenosine	reduced transmission via presynaptic A_1_	[53,54]
	GABA_B_	--	heterosynaptic depression requiring signaling at both A_1_ and NMDA receptors	[55]
	GABA_B_	glutamate	suppressed synaptic activity via presynaptic mGluR2/3	[56]
	--	D-serine	LTD induction during low frequency stimulation	[57]
	GLT-1	--	reduced magnitude of presynaptic LTP	[58]
	--	ATP/adenosine	synaptic depression via presynaptic P2Y receptors	[59]
	--	ATP/adenosine	AMPAr internalization via postsynaptic P2XRs	[60]
	--	--	reduced readily releasable vesicle pool via sybII+ SNARE-dependent vesicle release	[61]
	CB1	--	AMPAr internalization	[62,63]
	--	glutamate	AMPAr internalization	[64]
	GABA_B_	glutamate	synaptic potentiation via presynaptic mGluR1/5	[65,66]
	--	glutamate	potentiated transmitter release via presynaptic mGluR1/5	[67,68]
	P2Y1	glutamate	potentiated transmitter release	[69]
	CB1	glutamate	NMDAr-dependent SICs	[70]
	GLT-1	--	impaired mGluR-dependent LTD	[58]
	mAChR	glutamate	LTP via mGluR1/5	[71]
	CBr	--	LTP via coincident astroglial eCB detection, postsynaptic NO production, and presynaptic mGluR1/5 stimulation	[72]
	--	--	increased readily releasable vesicle size via ceb+ SNARE-dependent vesicle release	[61]
	CB1	D-serine	LTP	[73]
	VRAC	glutamate	synaptic potentiation via presynaptic mGluR1/5 and postsynaptic NMDArs	[74]
**Striatum**	System xc-	glutamate	synaptic depression via presynaptic mGluR2/3	[28]
	--	ATP/adenosine	synaptic depression via presynaptic A_1_	[53,54]
	CBr	--	LTD	[75]
	--	ATP/adenosine	LTD	[75]
	GLT-1	--	impaired temporal contingency required for spike timing-dependent LTP	[76]
	D_1_	ATP/adenosine	synaptic depression via presynaptic A_1_	[77]
	CB1	glutamate	synaptic potentiation via postsynaptic NMDArs and presynaptic mGluR1/5	[78]
	μ-opioid receptor	glutamate	NMDAr-dependent SICs	[79]
**Amygdala**	--	ATP/adenosine	synaptic depression via presynaptic A_1_	[80]
	CB1	--	AMPAr internalization	[81]
	--	ATP/adenosine	synaptic potentiation via presynaptic A_2a_	[80]
**Hypothalamus**	CB1	ATP/adenosine	synaptic depression via presynaptic A_1_	[82]
	α1 adrenoceptor	ATP/adenosine	AMPAr insertion via neuronal P2X7	[83]
	--	D-serine	NMDAr-dependent LTP	[84]
	mGluR	ATP/adenosine	postsynaptic purinergic receptors that increase neural activity	[85]
**Midbrain**	GLT-1	--	impaired CB1- and NMDAr-dependent LTD	[86]
**Brainstem**	--	ATP/adenosine	increased neural activity in response to low pH	[87]

**Table 2:** Diversity of reported astroglial responses to G_q_ signaling in adult brain. Note that the main G_q_-coupled receptor listed here mGluR5 has been shown to be developmentally regulated in brain astrocytes, and to be expressed at low levels in adulthood [97]. However, mGluR5 also appears to be functionally relevant, even at low levels and relatively plastic, increasing expression in various brain regions in response to local environmental cues [98]. Colors correspond to brain regions shown in the top panel where data were collected. * Conducted using human tissue samples, otherwise all reported studies were conducted using rodents. PAP, perisynaptic astroglial process; TBI, traumatic brain injury; SIC, slow inward current; EPSC, excitatory post-synaptic current; IPSC, inhibitory post-synaptic current; LTP, long-term potentiation; BLA, basolateral amygdala; mPFC, medial prefrontal cortex.

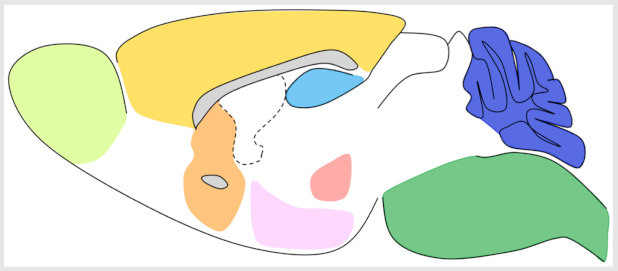				
Brain Region	Receptor	Molecular/Structural Effect	Model/Behavior	Ref.
Olfactory bulb	hM3Dq	↓ neuronal activity	↑ odor detection	[99]
Cortical cultures	mGluR5	↑ p-ezrin, filopodia motility	--	[31]
Cortical cultures	mGluR5	↑ ATP/adenosine release	Surrounding amyloid plaques	[100]
Cortical cultures *	mGluR5	↓ GLT-1 expression	--	[101]
Cortex	mGluR1/5	↑ vasodilation	--	[102]
Somatosensory cortex	mGluR5	↑ release of synaptogenic molecules	↑ mechanical allodynia	[103,104]
Barrel cortex	mGluR1/5	↑ PAP calcium	During whisker stimulation	[105]
Somatosensory cortex	hM3Dq	↑ slow-wave delta activity in neurons	--	[106]
Prefrontal cortex	hM3Dq	--	A_1_-dependent ↑ ethanol drinking (in naïve mice only) ↑ locomotor activation following low-dose ethanol ↑ sedative-hypnotic effects of high dose ethanol	[107]
Anterior cortex	Optoα1AR	↓ synaptic transmission via presynaptic A_1_	↓ locomotion ↑ long-term object recognition memory	[108]
Cortex	hM3Dq	↓ astroglial Ca^2+^	↓ sleep-wake transitions	[109]
Not stated	group I/II mGluRs	↑ vasoconstriction	--	[110]
Corpus callosum	mGluR5	↑ BDNF release	↑ myelination following cuprizone demyelination	[111]
Striatal cultures	mGluR5	↑ astrocyte swelling	--	[112]
Nucleus accumbens core	hM3Dq	--	↑ extinction of ethanol conditioned place preference	[113]
Nucleus accumbens core	hM3Dq	↑ SNARE-dependent glutamate release	↓ cued relapse	[94]
Nucleus accumbens core	hM3Dq	↑ synaptic adjacency	--	[9]
Nucleus accumbens core	hM3Dq	--	↓ motivation to obtain alcohol ↑ intracranial self-stimulation	[114]
Dorsolateral striatum	hM3Dq	↑ ATP/adenosine release ↓ synaptic transmission via presynaptic A_1_	--	[54]
Dorsolateral striatum	hM3Dq	↓ GLT-1	↑ behavioral flexibility	[115]
Dorsomedial striatum	hM3Dq	↓ transmission at synapses of the direct pathway (ENT1-dependent effect)	↓ habitual reward seeking	[116]
Dorsal striatum	hM3Dq	--	↑ locomotion	[117]
Hippocampus	mGluR5	↑ GAT-3 expression ↓ extracellular GABA	↓ TBI-induced cognitive dysfunction	[118]
Cortex				
CA1	mGluR5	↑ glutamate release ↑ neuronal SICs	--	[119,120,121]
Nucleus accumbens				
Hippocampal cultures	P2Y1	↑ filopodia formation	--	[122]
CA1	mGluR5	↑ excitatory transmission	↓ depressive-like behavior	[123]
CA1	mGluR5	↑ ATP/adenosine release ↑ synaptic transmission via presynaptic A_2A_	--	[124]
CA1	mGluR5	↑ ATP/adenosine release ↓ synaptic transmission via presynaptic A_1_	--	[53]
CA1	mGluR5	↑ glutamate uptake	Only after kainate-induced status epilepticus	[125,126]
CA1	mGluR5	↑ glutamate release ↓ neuronal SICs	Only after rapid kindling	[127]
Hippocampus	hM3Dq	↑ EPSC and IPSC amplitude and frequency	↑ contextual memory ↑ object recognition memory	[128]
CA1	hM3Dq	↑ neuronal firing ↑ neuronal SICs	--	[106]
CA1	hM3Dq	↑ NMDA-dependent LTP	↑ memory recall	[129]
Basolateral amygdala	hM3Dq	↑ synchronized neural activity in BLA and mPFC	↑ cued fear	[130]
Basolateral amygdala	hM3Dq	↑ extracellular glutamate	↓ ethanol consumption	[131]
Ventromedial hypothalamus	hM3Dq	--	↑ stress-induced anxiety and bone loss	[132]
Cerebellum	α_1A_ adrenergic receptor	↑ astroglial Ca^2+^	↑ behavioral vigilance	[133]
Ventral periaqueductal gray	hM3Dq	--	↑ sleep latency ↓ novelty-induced locomotion	[134]
Brainstem	hM3Dq	↑ morphological complexity	↓ feeding	[135]
